# Vascular Programs in Emerging and Re-Emerging RNA Virus Infections: Comparative Lessons from SARS-CoV-2, Flaviviruses, and Hantaviruses

**DOI:** 10.3390/v18101160

**Published:** 2026-10-19

**Authors:** Silvia Beltrami, Giovanni Strazzabosco, Giovanna Schiuma, Sabrina Rizzo, Marcello Baroni, Daria Bortolotti

**Affiliations:** 1 Department of Environmental and Prevention Sciences, University of Ferrara, 44121 Ferrara, Italy; silvia.beltrami@unife.it (S.B.); giovanni.strazzabosco@unife.it (G.S.); giovanna.schiuma@unife.it (G.S.); sabrina.rizzo@unife.it (S.R.); 2 Department of Life Sciences, University of Ferrara, 44121 Ferrara, Italy

**Keywords:** emerging RNA viruses, endothelial dysfunction, viral coagulopathy, immunothrombosis, thromboinflammation, vascular leak, platelets, complement, NETosis

## Abstract

Emerging and re-emerging RNA viruses frequently present with clinical phenotypes that extend beyond classical organ tropism and include vascular leakage, platelet dysregulation, coagulation imbalance, microvascular injury, and thromboinflammation. SARS-CoV-2, flaviviruses, and hantaviruses provide three complementary models of virus-induced vascular disease. This narrative review compares these viruses through a shared endothelial–coagulative framework and proposes four partially overlapping vascular programs: endothelial activation, endothelial barrier failure, coagulation activation, and platelet quantitative or qualitative dysfunction. These programs are intended as a conceptual synthesis of convergent clinical, pathological, and experimental evidence rather than as a quantitative classification system. The framework is used not only to organize known mechanisms, but also to generate testable predictions for cross-virus biomarker panels, organ-specific endothelial models, and phenotype-guided clinical studies. Severe COVID-19 illustrates inflammation-driven endothelial activation, platelet–leukocyte interactions, complement amplification, NETosis, and immunothrombosis. Within the flavivirus group, DENV is used as the principal model for systemic vascular leakage, thrombocytopenia, and hemorrhagic risk; selected non-dengue flaviviruses illustrate how shared endothelial, platelet, and coagulation modules are redirected toward placental dysfunction (ZIKV), neuroinvasion (WNV and JEV), or hepatic failure and coagulopathy (YFV). Hantavirus infections provide a distinctive model of capillary leak syndrome in which non-lytic endothelial infection, immune-mediated injury, and permeability signaling converge on renal and pulmonary microvascular beds. A comparative view suggests that thrombotic, hemorrhagic, and leaky phenotypes are not mutually exclusive categories, but different outcomes of overlapping vascular programs shaped by viral tropism, vascular-bed specialization, immune activation, and host susceptibility.

## 1. Introduction

The clinical impact of emerging RNA viruses is increasingly understood through the lens of vascular biology. Respiratory failure, hemorrhagic syndromes, renal dysfunction, shock, and neurological disease are often accompanied by endothelial activation, changes in vascular permeability, platelet consumption or activation, complement amplification, and coagulation imbalance. These processes do not merely represent secondary consequences of severe systemic inflammation. In several viral infections, they appear early, correlate with severity, and contribute directly to organ injury.

SARS-CoV-2, flaviviruses, and hantaviruses provide a useful comparative framework. SARS-CoV-2 brought unprecedented attention to viral immunothrombosis, in which epithelial and innate-immune sensing is amplified by cytokine release, endothelial activation, vWF secretion, tissue-factor induction, platelet–leukocyte interactions, complement activation, NET formation, and suppressed fibrinolysis. These coupled processes span a continuum from adaptive local immunothrombosis to pathological pulmonary microthrombosis and systemic venous or arterial thrombosis [1,2,3,4,5,6,7,8,9,10,11]. Flaviviruses, particularly dengue virus (DENV), have long been associated with thrombocytopenia, vascular leakage, and hemorrhagic manifestations. In dengue, NS1-mediated glycocalyx and barrier effects are supported by multiple mechanistic studies, whereas their magnitude in patients and extrapolation to other flaviviruses remain context-dependent [12,13,14,15,16]. Pre-existing immunity and host inflammatory responses further shape the vascular phenotype. Hantaviruses, transmitted from rodent reservoirs, cause hemorrhagic fever with renal syndrome (HFRS) and hantavirus cardiopulmonary syndrome (HCPS), both characterized by thrombocytopenia and abrupt capillary leakage in organ-specific microvascular beds [17,18,19,20,21].

The aim of this review is to synthesize endothelial and coagulative alterations induced by SARS-CoV-2, flaviviruses, and hantaviruses and to identify the shared and divergent mechanisms that shape clinical phenotypes. These three groups were selected because they represent clinically relevant and mechanistically complementary models along a vascular spectrum that includes thrombosis, hemorrhage, and capillary leak. The review does not attempt to cover all RNA viruses with vascular manifestations; instead, it uses these models to build a comparative framework that may be extended to other emerging infections. Filoviruses and arenaviruses were not included as co-equal index groups because the review was designed around three infections for which a sufficiently aligned human biomarker, pathology, and mechanistic data permit a comparison across all four programs; including Ebola, Marburg, and Lassa within the same depth would have required a separate systematic treatment of viral hemorrhagic fevers. Their exclusion is therefore a scope decision, not an assertion that the framework is inapplicable to them.

This narrative review focuses on pathogenesis and translational implications while highlighting knowledge gaps that may be addressed by endothelial models, organ-on-chip systems, omics technologies, and harmonized biomarker studies.

This review extends the prior comparative work on viral hemorrhagic-fever vascular biology, viral coagulopathy, and thromboinflammation [6,22,23] by placing SARS-CoV-2, dengue-centered flavivirus evidence, and hantaviruses within one endothelial–coagulative framework. Its distinct contribution is to treat endothelial activation, barrier failure, coagulation activation, and platelet dysfunction as overlapping, testable programs rather than fixed virus-specific categories.

The practical premise is that a patient, tissue model, or viral syndrome may be assigned to a dominant or mixed vascular program using convergent clinical variables and biomarkers. For example, a thromboinflammatory profile would be expected to show prominent coagulation activation, platelet–leukocyte interactions, NETosis, and endothelial activation markers, whereas a leaky profile would be expected to show barrier and glycocalyx disruption, hemoconcentration or tissue-specific edema, and only variable thrombin generation. This distinction is intended to guide research design rather than to replace virus-specific clinical judgment.

## 2. Review Strategy

This is a narrative review designed to provide a mechanistic and translational comparison of SARS-CoV-2, flaviviruses, and hantaviruses as complementary models of viral vascular disease. The objective was not to perform a systematic review or meta-analysis, but to synthesize shared and divergent mechanisms linking RNA virus infection to endothelial dysfunction, coagulation imbalance, platelet dysregulation, vascular leakage, and immunothrombosis.

Targeted searches of PubMed, Web of Science Core Collection, and Google Scholar were last updated on 31 July 2026 and were complemented by backward and forward citation tracking. Complete database-specific search strategies and the Google Scholar screening procedure are provided in Supplementary Methods S1. Searches were restricted to English-language, peer-reviewed articles; preprints and other non-peer-reviewed reports were used only for horizon scanning and were not used to classify a mechanism as established. No preprint supports a central conclusion of the present review.

Because the iterative narrative searches and citation-chaining updates were not prospectively logged, a defensible count of all records screened cannot be reconstructed retrospectively; 72 sources were retained and discussed in depth in the revised manuscript. This limitation is reported explicitly rather than presenting a back-calculated PRISMA-equivalent flow. Priority was given to human pathology, longitudinal clinical cohorts, and mechanistic studies with vascular or hemostatic endpoints; articles in which vascular findings were peripheral or indistinguishable from nonspecific critical illness were excluded from detailed synthesis.

Formal risk-of-bias scoring and pooled evidence grading were not performed. At least two co-authors reviewed each mechanism against the study type, reproducibility, disease specificity, and relevance of the vascular model; disagreements were resolved by discussion and, when a consensus was not reached, by a third co-author, with the more conservative evidence tier retained. Convergent human pathology and clinical data received the greatest weight; single cohorts, indirect biomarkers, non-human systems, and group-specific pilot findings remained context-dependent or hypothesis-generating.

Figure 2 assignments integrated clinical prominence, consistency across independent studies, and disease or vascular-bed specificity. Because these criteria still require judgment, the figure is explicitly labelled an expert schematic; Supplementary Table S1 provides, for every program × virus cell, the weight, supporting study types, key endpoints, and a one-line rationale so that readers can audit the interpretation.

SARS-CoV-2, flaviviruses, and hantaviruses were selected because they represent three informative and partially overlapping vascular disease models: thromboinflammatory vasculopathy, platelet–endothelial imbalance with hemorrhagic/leaky manifestations, and organ-specific capillary leak. Within the flavivirus group, dengue virus was treated as the principal model for systemic vascular leakage because it provides the strongest clinical and mechanistic evidence for plasma leak, thrombocytopenia, and hemorrhagic risk. Other flaviviruses were included selectively to show how shared vascular modules are redirected by organ tropism and specialized endothelial barriers toward neuroinvasion, placental dysfunction, or hepatic failure and coagulopathy. Other RNA viruses with vascular manifestations were considered relevant for context, but were not the primary focus of the comparative framework.

### 2.1. Interpretation of Evidence Strength

Throughout the review, evidence is interpreted at three levels. Established mechanisms refer to observations supported by multiple independent clinical, pathological, or mechanistic studies and consistently linked to a vascular phenotype. Supported but context-dependent mechanisms refer to findings reproduced in selected diseases, cohorts, viral species, disease stages, or experimental systems, but not yet generalizable across the entire viral group. Hypothesis-generating mechanisms refer to observations derived from limited cohorts, pilot studies, indirect biomarker associations, or specialized experimental models. This distinction is particularly relevant for direct endothelial infection by SARS-CoV-2, NS1-dependent effects across different flaviviruses, VEGF/integrin signaling in hantavirus permeability, and PROS1/protein S or HLA-G/NK/KIR-related observations in COVID-19 vascular disease. When recent or group-specific studies are cited to support emerging host-modifier pathways, they are interpreted as illustrative and hypothesis-generating unless independently reproduced in broader cohorts.

### 2.2. Framework Construction and Testable Predictions

Prediction 1 is that viruses with a different tropism converge on common downstream vascular modules, while patients with the same diagnosis diverge into dominant or mixed programs. Minimal readout: paired serial measurements of vWF or angiopoietin-2, syndecan-1, D-dimer plus thrombin-antithrombin complexes, platelet count plus platelet–leukocyte aggregates, aligned to adjudicated thrombosis, bleeding and tissue-edema outcomes.

Prediction 2 is that vascular-bed-specific models reproduce only the clinically relevant components of each syndrome. Minimal readout: transendothelial electrical resistance or tracer permeability together with VE-cadherin localization and a thrombin-generation assay in pulmonary, renal or glycocalyx-focused endothelial systems.

Prediction 3 is that program-guided stratification outperforms virus-only grouping. Minimal readout: prospective comparison of discrimination, calibration and reclassification for thrombosis, bleeding or leak using the multiparametric program panel versus viral diagnosis alone.

## 3. The Endothelial–Coagulative Axis in Viral Infection

The endothelium maintains the barrier integrity and antithrombotic tone through nitric oxide, prostacyclin, thrombomodulin, heparan sulfate, tissue factor pathway inhibitor, and the protein C-protein S-EPCR and antithrombin systems [24,25,26]. Viral infection can perturb these functions directly or, more commonly, through the cytokines, complement, circulating viral products, immune complexes, platelets, and metabolic stress [6,22].

Activated endothelium recruits leukocytes, releases vWF, supports tissue-factor-dependent thrombin generation, sheds glycocalyx, and suppresses fibrinolysis through PAI-1 [6,22,24,27]. Platelets then couple hemostasis to antiviral immunity through leukocyte aggregates, extracellular vesicles, and NET formation; accordingly, thrombocytopenia may coexist with intense platelet activation and a prothrombotic state [22,28,29].

The NETs and complement amplify this circuit by providing a scaffold for the platelets, vWF, tissue factor, and fibrin and by reinforcing endothelial and leukocyte activation [7,27,30]. The clinical output depends on vascular bed, timing, host reserve, and the relative dominance of barrier, coagulation, and platelet programs rather than on any single mediator.

**Figure 1:**
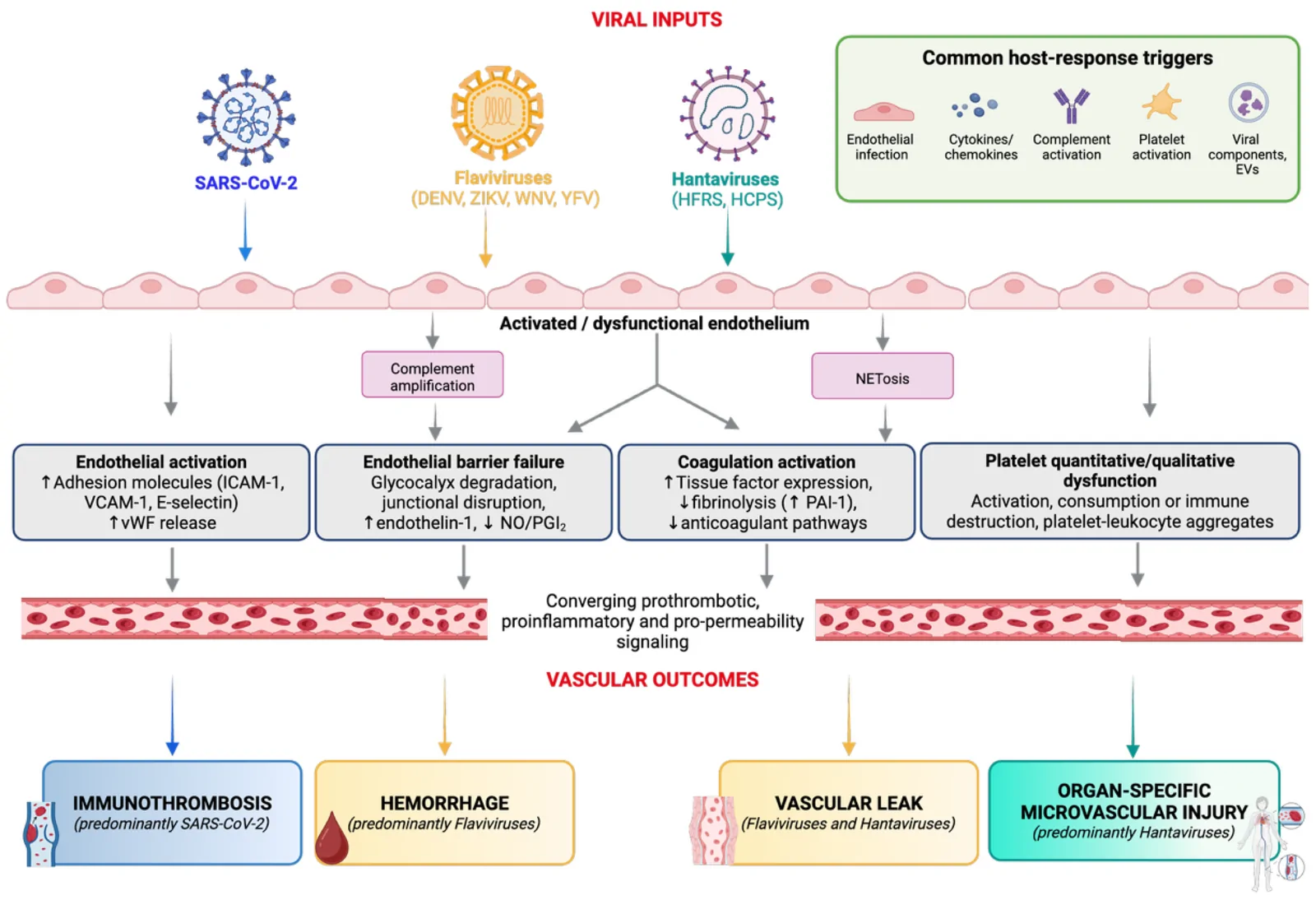
Viral vascular interface linking emerging RNA viruses and viral components to endothelial activation, glycocalyx disruption, vWF release, tissue factor expression, platelet and leukocyte activation, complement amplification, NETosis, and downstream clinical outputs, including immunothrombosis, hemorrhage, vascular leak, and organ-specific microvascular injury. For SARS-CoV-2, the scheme primarily represents indirect immune-mediated endothelial activation; the endothelial-infection route is context-dependent and does not imply widespread productive infection. Created in BioRender. Bortolotti, D. (2026) https://BioRender.com/5s0r9de (accessed on 12 October 2026).

Cross-virus comparisons must also account for major iatrogenic confounders: intensive-care admission, vasopressor exposure, volume resuscitation, mechanical ventilation or other invasive organ support, renal-replacement therapy, and prophylactic or therapeutic anticoagulation can independently alter permeability, platelet counts, D-dimer, thrombin-generation, and endothelial biomarkers. These exposures should be recorded as time-varying covariates rather than treated as background noise. The main link between viral components of emerging RNA viruses and endothelium are reported in Figure 1.

## 4. SARS-CoV-2: A Prototype of Inflammation-Driven Viral Immunothrombosis

COVID-19 changed the conceptual landscape of viral coagulopathy. Early in the pandemic, severe disease was recognized as a syndrome in which respiratory failure was accompanied by elevated D-dimer, high fibrinogen, increased vWF, endothelial activation, platelet hyperreactivity, and a high incidence of venous and arterial thrombotic complications [4,5,31]. Autopsy and histopathology studies described diffuse alveolar damage, microvascular thrombosis, endothelial injury, and angiogenic remodeling in lungs from patients with severe COVID-19 [2,3]. These observations supported the idea that SARS-CoV-2 produces a thromboinflammatory vasculopathy rather than a simple pneumonia complicated by nonspecific critical illness.

The mechanisms of SARS-CoV-2-associated coagulopathy are multifactorial. The viral infection of epithelial and immune cells initiates local inflammation, cytokine and chemokine production, the recruitment of neutrophils and monocytes, and endothelial activation [2,32]. The direct infection of endothelial cells in vivo remains debated and may vary according to vascular bed, disease stage, sampling site, and detection method. Accordingly, evidence of viral RNA or protein in vascular tissues should be distinguished from productive endothelial infection. Nevertheless, there is broad agreement that the endothelium is functionally injured in severe disease. Circulating markers of endothelial activation and damage, including vWF, soluble thrombomodulin, and angiopoietin-2, are associated with severity and adverse outcome in several cohorts [4,5]. Methodologically, RNA in situ hybridization combined with endothelial lineage markers can establish intracellular localization, whereas protein immunohistochemistry alone is more vulnerable to the cross-reactivity or uptake of extracellular material; neither demonstrates productive infection. The recovery of replication-competent virus, or convergent evidence of replicative intermediates and infectious progeny from authenticated endothelial cells, is required to support productive infection, and ACE2/TMPRSS2 expression in a relevant microvascular bed is permissive evidence but is not sufficient on its own.

Evidence cited in support of direct endothelial involvement includes endothelial-associated viral RNA or protein detected by in situ methods and reports of virus-like particles in vascular cells in autopsy material [1,3,33]. These findings support anatomical proximity or cellular uptake but do not, on their own, establish the completion of the viral replication cycle. Conversely, primary endothelial-cell, three-dimensional vessel and transcriptomic studies have reported a very low or heterogeneous ACE2/TMPRSS2 expression, inefficient infection except under high-titer or receptor-overexpression conditions, and no substantial production of infectious progeny [34,35]. Interpretation is further limited by the difficulty of excluding the signal from adjacent epithelial cells, pericytes, circulating virions, or phagocytosed material. Productive infection should therefore require convergent evidence of endothelial-cell identity, intracellular replicative intermediates, and the release of infectious progeny. Until such evidence is consistently reproduced across vascular beds, endothelial exposure or uptake may be considered possible in selected settings, but widespread productive endothelial infection is not required to explain COVID-19 vasculopathy.

Three recent studies from the authors’ group (2024–2026) describe PROS1/protein S and HLA-G/NK/KIR-HLA associations in selected SARS-CoV-2 vascular contexts [33,36,37]. These unreplicated observations are retained as hypothesis-generating modifiers and should be interpreted with concurrent vitamin K status, liver function, anticoagulant exposure, acute-phase state, and lipoprotein context; they are not universal or virus-specific markers. Irrespective of whether direct endothelial infection occurs in selected settings, the core evidence base for SARS-CoV-2-associated coagulopathy rests on systemic inflammation, complement activation, platelet–leukocyte crosstalk, NETosis, endothelial activation, elevated D-dimer, and altered fibrinolytic balance [2,4,5,8,9,10,27].

Platelet activation is a central feature of COVID-19-associated immunothrombosis [5,8]. Activated platelets interact with monocytes and neutrophils, promote tissue factor expression, release proinflammatory mediators, and contribute to microvascular obstruction. Platelet–leukocyte aggregates and platelet-derived extracellular vesicles can propagate coagulation activation. At the same time, neutrophils release NETs that provide a scaffold for thrombin generation and fibrin deposition. Complement activation further amplifies endothelial injury and leukocyte recruitment, creating a thromboinflammatory loop [1,8,9,10,27,32].

The canonical COVID-19-associated coagulopathy profile—marked D-dimer elevation with normal or high fibrinogen, high factor VIII/vWF, and relatively modest thrombocytopenia—was defined mainly during the first pandemic waves in unvaccinated cohorts, often before consistent ward-level thromboprophylaxis [38,39]. It differs from early consumptive DIC but may evolve toward falling fibrinogen, thrombocytopenia, and prolonged PT/aPTT in advanced critical illness. Vaccination and early antiviral treatment reduce the progression to severe systemic disease, while routine prophylaxis and earlier anticoagulant exposure alter the observed thrombosis rates and coagulation assays [40,41,42,43]; therefore, the first-wave laboratory phenotype should not be assumed to represent all patients in 2026 practice.

SARS-CoV-2 remains the benchmark for the thrombotic end of the spectrum. Small, heterogeneous follow-up cohorts have reported persistent endothelial or coagulation abnormalities after acute infection, but variable case definitions, survivor selection, absent pre-infection baselines, and changing vaccination or treatment exposure prevent the assignment of a distinct post-acute vascular program [44].

## 5. Flaviviruses: Vascular Leakage, Platelets, and the Hemorrhagic Balance

Flaviviruses are enveloped, positive-sense RNA viruses transmitted mainly by arthropods. Clinically relevant human flaviviruses include DENV, Zika virus (ZIKV), yellow fever virus (YFV), West Nile virus (WNV), Japanese encephalitis virus (JEV), tick-borne encephalitis virus (TBEV), and Usutu virus (USUV). They differ in organ tropism and epidemiology, but several members of the genus interact with the vascular interface through endothelial activation, platelet dysregulation, and barrier disruption [45,46,47].

DENV is the most informative model for flavivirus vascular disease. Severe dengue is characterized by plasma leakage, hemoconcentration, thrombocytopenia, bleeding, and, in some cases, shock and organ dysfunction [45,47]. The vascular leak is typically transient and can develop around defervescence, suggesting that the host immune responses and soluble mediators, rather than uncontrolled endothelial cytolysis, are major drivers. The secreted viral protein NS1 is a well-supported virulence factor in DENV experimental systems. Multiple independent mechanistic studies show that NS1 can bind endothelial cells, disrupt components of the endothelial glycocalyx, trigger inflammatory pathways, and promote barrier dysfunction [12,13,14,48,49]. NS1-dependent glycocalyx and barrier disruption can therefore be considered an established mechanism in experimental dengue models; however, its quantitative contribution in patients remains context-dependent and should not be generalized to all flaviviruses.

The evidentiary layers are distinct: purified NS1 and infection experiments demonstrate concentration- and serotype-dependent glycocalyx injury in endothelial systems and animal models [12,13,48], whereas human cohorts show associations among circulating NS1, syndecan-1, or other glycocalyx fragments and plasma-leak severity without proving that NS1 alone caused the leak [50,51]. NS1-dependent injury may therefore be a necessary component in some dengue contexts, but it is not sufficient for clinical leakage, which also depends on the immune history, cytokines, platelets, vascular bed, and disease timing.

**Figure 2:**
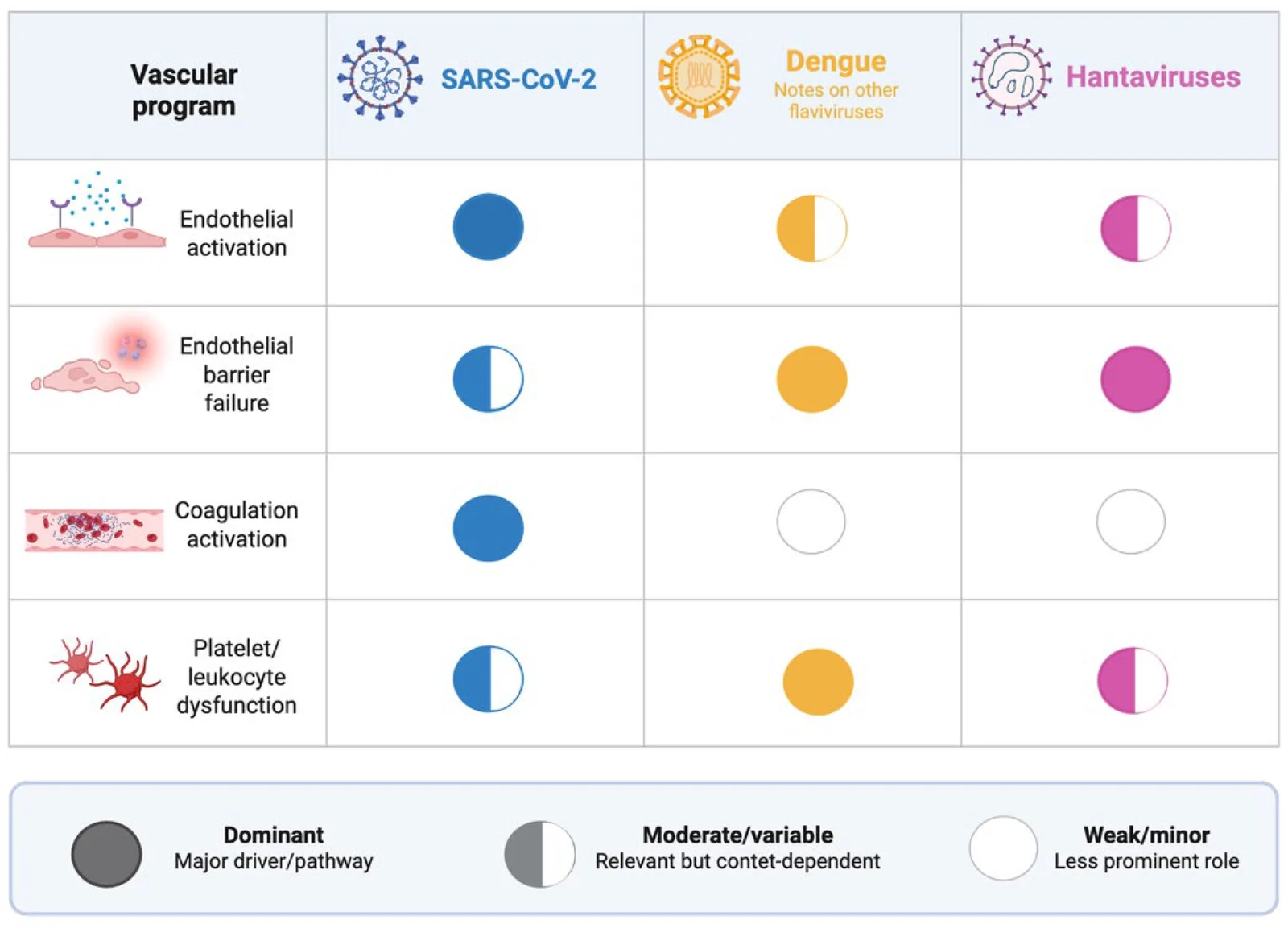
Qualitative weighting of the four proposed vascular programs across SARS-CoV-2, flaviviruses, and hantaviruses. The flavivirus weighting is driven primarily by DENV and should not be extrapolated uniformly to neurotropic, placentotropic or viscerotropic flaviviruses (reported as “Dengue (with notes on other flaviviruses)”). Filled circles indicate programs that are dominant in the typical severe phenotype, half-filled circles indicate moderate or context-dependent contributions, and open circles indicate weak, variable, or less consistently documented contributions. The diagram integrates clinical prominence, mechanistic support, and disease specificity. It should therefore be interpreted as an expert schematic, not a quantitative ranking: the circle patterns summarize author consensus regarding the relative prominence of each vascular program and do not represent numerical scores, pooled estimates, formal evidence grades, effect sizes, or ranks. Individual patients, viral species, vascular beds, and disease stages may deviate from this simplified pattern. Cell-level evidence and rationales are reported in Supplementary Table S1. Created in BioRender. Bortolotti, D. (2026) https://BioRender.com/cyvof45 (accessed on 12 October 2026).

Beyond endothelial permeability, a role for NS1 in coagulation activation is less certain and should not be generalized from dengue to the flavivirus genus. A pilot study from the authors’ group reported the virus-specific modulation of tissue factor-bearing microvesicle release: NS1 from West Nile and Japanese encephalitis viruses increased procoagulant activity, whereas DENV NS1 showed an anticoagulant effect associated with differential IL-6 modulation [52]. These findings are hypothesis-generating and currently unreplicated; they do not establish a conserved NS1-coagulation mechanism, are not used to determine Figure 2 weighting or support clinical recommendations, and require validation in independent cellular systems, tissue-relevant endothelial models, and patient cohorts.

Thrombocytopenia in dengue is multifactorial. Mechanisms include bone marrow suppression, immune-mediated platelet destruction, platelet activation, consumption, and interactions with the complement and the endothelium [29,53]. Platelets from patients with dengue can display activation markers and contribute to inflammatory signaling despite reduced numbers. Consequently, platelet count alone is an incomplete surrogate for bleeding risk. The severity of vascular leakage and the dynamics of coagulation and fibrinolysis are equally important for the clinical outcome [53,54]. In severe dengue, coagulation abnormalities may include the prolongation of PT and/or aPTT, reduced fibrinogen, increased fibrin turnover, and the consumption or reduced synthesis of coagulation factors, especially when hepatic involvement, shock, or severe plasma leakage are present. Thus, the bleeding phenotype reflects the convergence of thrombocytopenia, endothelial leak, platelet dysfunction, and coagulation-factor imbalance rather than platelet count alone. Accordingly, a low platelet count should not be equated automatically with hypocoagulability; immature platelet fraction, platelet activation, or function assays and platelet–leukocyte aggregates may help distinguish impaired production from activated consumption, although they remain insufficiently standardized for routine dengue decisions.

Dengue also illustrates the complexity of antibody-mediated disease enhancement. Secondary infection with a heterologous serotype can increase the risk of severe disease through antibody-dependent enhancement and altered immune activation [16,47]. This may increase the viral burden and inflammatory mediator release, indirectly aggravating endothelial dysfunction. However, severe dengue cannot be reduced to one mechanism; viral genotype, NS1 properties, host genetics, age, prior immunity, and comorbidities all shape the vascular phenotype.

The same vascular modules can produce different organ-specific outcomes in non-dengue flavivirus infections. ZIKV targets placental, neural, and endothelial compartments; barrier perturbation at the maternal–fetal interface may promote placental dysfunction and fetal exposure, while neurovascular involvement contributes to neurological disease rather than generating the systemic plasma-leak phenotype typical of severe dengue [55]. In WNV and JEV infection, endothelial activation, blood–brain barrier dysfunction, and neurovascular inflammation may facilitate leukocyte trafficking and viral entry into the central nervous system [56]. Here, the dominant vascular consequence is neuroinvasion, not generalized hemorrhagic leakage. YFV provides a distinct viscerotropic pattern: hepatic injury and systemic endothelial activation converge with coagulation-factor depletion or consumption to produce coagulopathy and hemorrhage [57], so bleeding does not simply reproduce dengue-associated platelet/barrier dysfunction. TBEV and USUV further support the neurovascular pattern among selected flaviviruses with neuroinvasive potential [58]. These organ-specific vascular links are supported but context-dependent because their evidentiary strength varies among viruses and often derives from specialized experimental models or selected clinical cohorts. Thus, endothelial barrier disruption, platelet/hemostatic disturbance, and inflammation are shared modules, but their clinical expression is redirected by tissue tropism and vascular-bed specialization.

Accordingly, dengue is treated as the strongest model for systemic flavivirus vascular leakage; ZIKV for placental and neurovascular dysfunction; WNV, JEV, TBEV, and USUV for blood–brain-barrier-centered neuroinvasion; and YFV for the intersection of hepatic failure, endothelial activation, and systemic coagulopathy. This virus-specific mapping prevents a common vascular vocabulary from being interpreted as a uniform flavivirus disease mechanism.

Compared with SARS-CoV-2, the flavivirus vascular phenotype is generally less dominated by macrovascular thrombosis and more by thrombocytopenia, barrier dysfunction, and bleeding risk. However, this statement applies most directly to dengue and yellow fever; neurotropic flaviviruses more often illustrate the dysfunction of specialized endothelial barriers such as the blood–brain barrier. Coagulation activation can occur across flavivirus infections, and severe disease may include hemorrhagic manifestations, microvascular dysfunction, and inflammatory coagulopathy. In this sense, flaviviruses are not simply hemorrhagic viruses, but vascular pathogens that alter the platelet–endothelial–coagulation balance in a virus- and tissue-specific manner.

## 6. Hantaviruses: Capillary Leak and Organ-Specific Microvascular Injury

Hantaviruses are enveloped, negative-sense RNA viruses maintained in rodent, shrew, mole, or bat reservoirs, with humans typically infected through the inhalation of aerosolized excreta. Pathogenic hantaviruses cause two overlapping clinical syndromes: HFRS, classically associated with Old World hantaviruses such as Hantaan, Seoul, Dobrava-Belgrade, and Puumala viruses and characterized by renal involvement, and HCPS, classically associated with New World hantaviruses such as Sin Nombre and Andes viruses and characterized by pulmonary edema with cardiopulmonary compromise [17,18,19,20,21,59,60]. Although these categories are useful, clinical manifestations can overlap, and both syndromes are fundamentally vascular diseases.

For the purposes of vascular comparison, HFRS and HCPS can be viewed as organ-weighted expressions of a shared permeability program. In HFRS, renal microvascular dysfunction is reflected by proteinuria, hematuria, impaired filtration, and fluid-balance disturbances, often with thrombocytopenia and inflammatory activation [59,60]. In HCPS, the same broad endothelial-permeability logic is concentrated in the pulmonary microvasculature, producing rapidly progressive non-cardiogenic pulmonary edema, hypoxemia, and shock [61]. This organ specificity emphasizes that viral vascular disease depends not only on the pathway being activated, but also on the endothelial phenotype and hemostatic tone of the affected vascular bed.

A striking feature of hantavirus disease is severe vascular permeability without the extensive endothelial cell destruction expected if cytopathic injury were the primary mechanism [17,61]. Hantavirus infection of endothelial cells, particularly in pulmonary and renal microvascular beds, is well-supported and is generally considered non-lytic [17,21]. The inference that abrupt permeability results from immune-mediated endothelial dysfunction and altered junctional signaling is supported by convergent studies. By contrast, the specific contributions of integrin signaling, VEGF pathway modulation, and the kallikrein-kinin/bradykinin pathway are supported but context-dependent because much of the evidence derives from particular hantavirus species, vascular beds, or experimental systems [18,19,21,62]. Their relative contribution to human HFRS and HCPS remains incompletely established.

Human HCPS autopsy series show abundant hantaviral antigen or RNA in the pulmonary microvascular endothelium with severe edema but preserved endothelial and alveolar architecture and little cytopathic destruction [23,63,64]. Native-tissue evidence is the strongest for New World HCPS; Old World HFRS renal biopsies support endothelial-centered inflammation and relative structural preservation, but the generalization of strictly non-lytic infection across species still relies substantially on cultured endothelial systems [59,60,65]. This pattern contrasts with filovirus disease, in which the infection of mononuclear phagocytes and multiple parenchymal compartments, tissue-factor-driven coagulopathy, and more destructive multiorgan pathology are prominent [66].

The strongest mechanistic support for the hantavirus capillary leak comes from the convergence of non-lytic endothelial infection, platelet–endothelial interactions, permeability-factor signaling, and immune-mediated injury. The multicomponent model is therefore supported, but assigning dominant causal weight to an individual pathway across all hantaviruses remains hypothesis-generating. These pathway-specific predictions require validation in independent human cohorts and organ-relevant endothelial models. Comparative studies that align HFRS and HCPS samples by time from symptom onset, platelet dynamics, renal or pulmonary leak markers, and endothelial activation markers would help determine whether these syndromes represent distinct programs or organ-weighted variants of a shared leak pathway.

Thrombocytopenia is a hallmark of hantavirus infection and often appears early [29,67]. Platelets may interact with infected endothelial cells and viral glycoproteins, become activated or sequestered, and participate in immune-endothelial signaling. In HFRS, thrombocytopenia accompanies renal endothelial dysfunction, proteinuria, hematuria, and changes in vascular permeability [59,60]. In HCPS, a pulmonary capillary leak leads to non-cardiogenic pulmonary edema, hypoxemia, and shock [61]. Coagulation abnormalities, including prolonged clotting times and elevated D-dimer, can be observed, but the dominant clinical problem is often leakage rather than thrombosis or bleeding alone. Although macrovascular thrombosis is not the defining feature, hantavirus infection should not be interpreted as hemostatically inert. Hemoconcentration, endothelial activation, thrombocytopenia, renal dysfunction, and variable thrombin generation may coexist, making the bleeding and thrombosis risk highly dependent on the disease phase and organ involvement. As in dengue, a low count can coexist with platelet activation and thrombin generation; immature platelet fraction and standardized platelet-function or aggregate assays should be incorporated into prospective cohorts rather than inferring the net hemostatic state from count alone.

The host immune response is central to hantavirus pathogenesis. Robust cytotoxic T-cell responses, cytokines, monocyte/macrophage activation, and complement can contribute to endothelial barrier dysfunction [17,61]. At the same time, effective antiviral immunity is necessary for viral control. The challenge is therefore not to suppress immunity indiscriminately, but to understand which immune-endothelial circuits drive permeability and organ injury. Comparative studies of mild and severe hantavirus infections may be particularly informative because they can distinguish protective from pathogenic vascular immune responses [18,61].

In the comparative framework of this review, hantaviruses represent the leaky end of the viral vascular spectrum. They demonstrate that dramatic clinical vascular permeability can arise with limited direct cytopathic damage and without a primary macrothrombotic phenotype. This distinguishes hantavirus disease from COVID-19 while linking it mechanistically to aspects of dengue, especially transient endothelial barrier failure and thrombocytopenia.

## 7. Comparative Pathogenesis: From Thrombosis to Hemorrhage and Leak

The three viral groups reviewed here converge on the endothelial–coagulative axis but diverge in clinical output. SARS-CoV-2 tends toward thromboinflammatory occlusion [2,8], dengue toward platelet/endothelial imbalance with bleeding and leak [53,54], and hantaviruses toward organ-specific capillary leakage [17,61]. These phenotypes are not mutually exclusive. Severe dengue can include coagulation activation, COVID-19 can include endothelial barrier failure, and hantavirus disease can include hemorrhagic manifestations and coagulation abnormalities. In other flaviviruses, these modules are redistributed toward organ-specific neurovascular, placental, or hepatic phenotypes. The key question is why the balance differs.

Several variables likely determine the dominant phenotype. First, viral tropism and viral products matter. NS1-mediated endothelial glycocalyx disruption is established in experimental DENV systems but remains context-dependent in patients and cannot be extrapolated uniformly across flaviviruses [12,48]. SARS-CoV-2 pathogenesis is strongly shaped by respiratory infection, inflammatory amplification, and platelet–leukocyte interactions [2,5]. Hantavirus infection of endothelial cells is well-supported as predominantly non-lytic, whereas the relative contribution of specific VEGF/integrin permeability pathways remains context-dependent [19,21]. Second, the vascular bed matters. Pulmonary microvessels, renal glomerular and peritubular capillaries, hepatic sinusoids, the placental interface, and the blood–brain barrier differ in endothelial phenotype, hemostatic tone, and immune surveillance [24,68]. Third, host immunity matters. Age, prior flavivirus exposure, metabolic disease, genetic background, complement regulation, and baseline endothelial health can shift the response toward thrombosis, leak. or bleeding [16,47].

A useful conceptual model is to separate four partially overlapping programs: endothelial activation, endothelial barrier failure, coagulation activation, and platelet quantitative/qualitative dysfunction. SARS-CoV-2 strongly activates endothelial and coagulation programs with platelet activation, producing immunothrombosis [5,8]. Dengue strongly activates barrier failure and platelet dysfunction, with variable coagulation activation, producing leakage and hemorrhagic risk [53,54]. Hantavirus disease strongly activates barrier failure in selected microvascular beds, with thrombocytopenia and immune-mediated permeability [17,61]. This four-program framework is proposed as the central comparative contribution of this review. It is not intended to classify viruses permanently, but to generate testable hypotheses: patients with severe disease should separate into thrombotic, hemorrhagic, leaky, or mixed vascular phenotypes; biomarker panels should perform better when interpreted by program rather than by virus name alone; and tissue-specific endothelial models should reproduce different program combinations depending on vascular bed. To make the comparison explicit, Figure 2 summarizes the relative prominence of each vascular program in the dominant clinical phenotype associated with SARS-CoV-2, dengue-centered flavivirus evidence, and hantaviruses. This synthesis is conceptual and narrative rather than quantitative, and takes into account the strength, consistency, and disease specificity of the available evidence. It is intended to guide biomarker selection, experimental modeling, and therapeutic stratification. A patient with severe dengue plus independently documented thrombin generation would be classified as a mixed barrier-failure/platelet/coagulation phenotype, with the assigned programs updated serially rather than forced into one category.

The transfer to classical viral hemorrhagic fevers is plausible but not assumed. Ebola and Marburg would be expected to produce a mixed endothelial-activation, barrier-failure, coagulation and platelet phenotype in which monocyte/macrophage infection, tissue-factor expression, hepatic dysfunction, and multiorgan injury may outweigh the relatively non-lytic leak pattern of hantavirus disease [66]. Lassa fever may also map to a mixed program, but fatal-case pathology shows heterogeneous endothelial and mononuclear-phagocyte involvement rather than a single uniform vascular lesion [69]. These pathogens are therefore stringent prospective tests: the model would be supported only if program-level measurements predict the leak, bleeding, and thrombosis better than disease labels alone.

The endothelial and hemostatic profiles of SARS-CoV-2, dengue-centered flavivirus evidence, and hantaviruses are compared across ten prespecified indicators in Table 1. Evidence tiers are stated within individual cells to distinguish established, context-dependent, and hypothesis-generating claims.

## 8. Biomarkers of Endothelial Injury, Coagulation Imbalance, and Vascular Leak

Biomarkers are essential for translating vascular pathogenesis into clinical decisions. Routine tests such as platelet count, D-dimer, fibrinogen, prothrombin time, activated partial thromboplastin time, liver enzymes, creatinine, hematocrit, and lactate remain indispensable. However, they often cannot distinguish endothelial activation from barrier failure, platelet consumption from immune destruction, or controlled immunothrombosis from harmful microvascular occlusion. A practical biomarker strategy should therefore distinguish clinically available markers from exploratory endothelial, glycocalyx, complement, NETosis, and anticoagulant-pathway markers that require assay harmonization and prospective validation. In this context, global coagulation assays, including thrombin- or FXa-generation approaches, may help capture the net procoagulant potential of plasma beyond routine clotting times, although their application to viral vascular syndromes requires disease-specific validation [72]. Candidate biomarkers that may support vascular phenotyping across these infections are summarized in Table 2.

Endothelial biomarkers may improve phenotyping. vWF antigen and activity, soluble thrombomodulin, soluble E-selectin, angiopoietin-2, syndecan-1, and markers of glycocalyx shedding can reflect endothelial activation or injury [4,70]. In COVID-19, high vWF and angiopoietin-2 have been associated with disease severity [4,70]. In dengue and hantavirus infection, markers of glycocalyx disruption and permeability may help identify patients at risk for plasma leakage [48,50,54,61]. Complement components and activation fragments may identify patients in whom complement-endothelial crosstalk is prominent [27]. NET markers, including cell-free DNA, myeloperoxidase-DNA complexes, and citrullinated histone H3, may capture neutrophil-driven thromboinflammation [9,10]. At present, most of these biomarkers should be considered tools for vascular phenotyping and translational research rather than stand-alone clinical decision markers. Their interpretation requires standardized sampling, disease-stage annotation, and integration with routine clinical variables.

Protein S is included as an example of how endogenous anticoagulant pathways may connect endothelial biology, coagulation, and lipid-rich vascular environments [24,71]. Protein S is detectable on plasma lipoproteins, particularly LDL/VLDL fractions, and within atherosclerotic plaques, emphasizing the broader interface between coagulation, lipids, and vascular disease [71]. For viral vascular syndromes, PROS1/protein S measurements may be most informative in predefined subgroups, such as patients with pre-existing atherosclerosis, dyslipidemia, cardiovascular comorbidity, or thrombotic complications [33,71], rather than as a universal biomarker across all infections. The functional relevance of protein S membrane interactions is further supported by studies of natural protein S variants showing that altered phospholipid binding can impair anticoagulant activity, reinforcing the importance of the membrane-localized regulation of thrombin generation [26].

Serial sampling is essential because dengue leak clusters around defervescence, COVID-19 thromboinflammation evolves with respiratory deterioration, and hantavirus leak may develop abruptly [31,38,45,46,61]. A minimal shared schedule is as follows: T0, admission before antiviral, anticoagulant, major fluid or vasopressor exposure whenever feasible; T1, 24–48 h; T2, the biological transition point (dengue defervescence or ICU day 3 for COVID-19/hantavirus); and T3, early recovery or discharge, with a convalescent sample at 30–90 days when feasible. The exact time from symptom onset and all major interventions should accompany every specimen.

## 9. Therapeutic and Translational Implications

A vascular framework has therapeutic implications but does not imply one treatment across infections. In the multiplatform randomized trials, therapeutic-dose heparin improved organ-support-free survival in non-critically ill hospitalized patients, with more major bleeding, whereas it did not improve survival or organ-support-free days in critically ill patients and showed a high probability of futility or harm [40,41]. Dengue generally contraindicates empiric therapeutic anticoagulation when thrombocytopenia, bleeding risk, and plasma leakage dominate [45,46], and hantavirus management remains primarily supportive for shock, pulmonary edema, or renal failure [61].

The most plausible future interventions will be pathogenesis-informed and phenotype-specific. Antivirals administered early may reduce the upstream stimulus for endothelial activation. Endothelial-protective strategies, complement modulation, NET-targeting approaches, antiplatelet interventions, or anticoagulation may be relevant in selected settings, but they require careful timing and risk stratification. A coagulation-guided approach should integrate platelet count, bleeding signs, fibrinogen, PT/aPTT, D-dimer trajectory, renal and hepatic function, and anticoagulant exposure. The same pathway can be beneficial or harmful depending on context; for example, immunothrombosis may contribute to pathogen containment early but drive microvascular injury when excessive [6,7]. Therefore, the proposed vascular-program framework should be viewed as a hypothesis-generating tool for trial design and patient stratification, not as a direct therapeutic algorithm.

Operationally, the apparent contradiction is resolved by the dominant vascular program rather than by D-dimer alone. In a patient with COVID-19 and a coagulation-dominant phenotype, a rising D-dimer may contribute to the thrombotic risk assessment and, together with the disease stage, bleeding risk, and validated clinical criteria, support an established anticoagulation strategy [40,41]. In dengue, the same value may reflect the fibrin turnover during shock or plasma leak and, in a barrier-failure/platelet-dysfunction phenotype, should prompt the bleeding and leakage assessment rather than empiric therapeutic anticoagulation [45,46]. In hantavirus disease, D-dimer is currently indeterminate unless integrated with evidence of thrombosis, bleeding, thrombocytopenia, organ dysfunction, and capillary leak [61]. Thus, management is guided by the dominant or mixed vascular program, while remaining constrained by virus-specific evidence and not by the viral label or biomarker value in isolation.

A hypothetical longitudinal application illustrates this approach. In severe COVID-19, serial increases in vWF, D-dimer, and platelet–leukocyte aggregates with relatively stable syndecan-1 would support an endothelial-activation/coagulation-dominant phenotype and prompt thrombosis surveillance, while anticoagulation would continue to follow validated clinical criteria. In severe dengue, rising syndecan-1 and hemoconcentration with a falling platelet count, despite only modest changes in D-dimer or vWF, would support a barrier-failure/platelet-dysfunction phenotype and prioritize the assessment of leakage and bleeding. Concurrent increases in thrombin-generation markers would reclassify the dengue case as a mixed barrier/platelet/coagulation phenotype. These illustrative assignments should be updated at each sampling point and should not be used as stand-alone treatment triggers.

Accordingly, clinical trials and observational studies should incorporate vascular phenotyping. Instead of grouping patients only by viral diagnosis, studies could stratify by dominant vascular program: thrombotic, hemorrhagic, leaky, or mixed. Such stratification may identify subgroups in whom anticoagulation, complement modulation, endothelial stabilization, or supportive barrier-focused care is more likely to help. For emerging infections, preparedness protocols should include the standardized collection of samples for coagulation, endothelial, and immune biomarkers so that vascular mechanisms can be characterized early in outbreaks and translated rapidly into adaptive trial designs.

This comparison also generates testable predictions for clinical practice. First, multiparametric panels that combine endothelial activation, permeability, coagulation, and platelet signals should distinguish thrombotic, hemorrhagic, leaky, and mixed phenotypes more accurately than virus diagnosis or any single biomarker. Second, serial program assignment should predict changes in thrombosis, bleeding, or fluid-management risk and identify patients for whom adjunctive anticoagulant, barrier-focused, or investigational endothelial-directed strategies are more or less appropriate. Third, the framework should retain the predictive value when applied to future emerging infections with a different tissue tropism. Comparing SARS-CoV-2, flaviviruses, and hantaviruses therefore shifts the translational question from ‘which virus?’ to ‘which vascular program is dominant now?’, providing a virus-agnostic but disease-constrained model that can be prospectively validated in observational cohorts and adaptive trials.

Table 3 translates the proposed vascular programs into candidate study variables. This table is not a therapeutic algorithm; rather, it illustrates how the same viral diagnosis could be subdivided into biologically meaningful vascular phenotypes for observational cohorts, organ-on-chip experiments, or adaptive clinical trials. To make the mechanism-to-intervention link explicit, the expanded table also maps each program to a conditional therapeutic or management principle. These implications remain subordinate to viral diagnosis, disease stage, bleeding risk, organ function, and applicable clinical guidance; biomarker values alone should not trigger treatment escalation.

## 10. Future Perspectives

Major knowledge gaps remain. First, the degree to which each virus directly infects endothelial cells in relevant human vascular beds requires better definition. Second, endothelial responses differ across tissues; yet, many studies rely on generic endothelial cell lines or macrovascular cells. Third, the temporal sequence linking viral replication, soluble viral proteins, immune activation, platelet changes, the complement, and permeability is incompletely mapped. Fourth, many biomarkers are studied in single diseases, limiting the cross-virus comparison.

Future work should prioritize human microvascular endothelial models, co-cultures with platelets and leukocytes, organ-on-chip systems, vascularized organoids, and animal models that reproduce human vascular phenotypes. Single-cell and spatial transcriptomics can define infected and bystander cell populations in tissues. Proteomics and metabolomics can identify endothelial and coagulation signatures associated with progression. Importantly, studies should include both severe and mild cases, because mechanisms that distinguish protective vascular activation from pathogenic vascular collapse are likely to be most informative.

The factor XII-kallikrein-kinin axis is a specific unresolved priority in hantavirus disease. In endothelial models, factor XII-dependent plasma kallikrein activation can release bradykinin, activate B2-receptor signaling, and destabilize junctional barrier function [62]. Measuring FXII activation, kallikrein activity, cleaved high-molecular-weight kininogen, and bradykinin-pathway products in aligned HFRS/HCPS samples would test human relevance. FXII, plasma-kallikrein, C1-inhibitor, or bradykinin-receptor targeting is mechanistically plausible but remains investigational and must be evaluated against bleeding, thrombosis, and hypotension risks.

The prioritized research agenda is summarized in Table 4.

Priority 1—validate program assignment prospectively: first experiment, a shared four-time-point panel in aligned COVID-19, dengue and hantavirus cohorts measuring vWF/angiopoietin-2, syndecan-1, D-dimer/TAT, and platelet–leukocyte aggregates. Priority 2—define vascular-bed specificity: compare pulmonary, renal, and glycocalyx-focused microvascular endothelial models using permeability, VE-cadherin, and thrombin-generation readouts. Priority 3—test the glycocalyx as a shared final pathway: use a perfused organ-on-chip with matched patient plasma and quantify syndecan-1 shedding and tracer leak. Priority 4—resolve platelet state despite thrombocytopenia: pair immature platelet fraction with activation, aggregation, and leukocyte-binding assays. Priority 5—identify viral and host switches that redirect phenotype: perturb NS1, complement, NET, and VEGF/kallikrein pathways in the appropriate organ model. Priority 6—independently replicate the authors’ hypothesis-generating PROS1/HLA-G/KIR and non-DENV NS1 findings [33,36,37,52,71] in preregistered external cohorts or laboratories before they influence weighting or translation.

## 11. Conclusions

The four-program model would be refuted if prospectively measured endothelial-activation, barrier, coagulation, and platelet panels failed to improve the prediction of thrombosis, bleeding, or leak beyond viral diagnosis, severity, and treatment exposure, or if serial program changes did not track those outcomes. Nevertheless, even if such vascular panels prove informative, the central therapeutic priority remains the development and early use of virus-specific antiviral agents capable of suppressing viral replication, alleviating clinical manifestations and reducing progression to severe disease. In a new outbreak, the first deployable panel would combine platelet count and immature platelet fraction, D-dimer and fibrinogen, vWF or angiopoietin-2, syndecan-1, and platelet–leukocyte aggregates, collected on the shared schedule proposed above. The serial monitoring of endothelial activation, barrier integrity, coagulation, and platelet-related indicators, interpreted alongside virological findings, clinical status, and treatment exposure, could support timely risk stratification, guide treatment selection or adjustment, and help identify disease progression. However, these biomarkers should not independently trigger medication changes until their clinical utility has been prospectively validated. Because the current evidence base is narrative and heterogeneous, it cannot provide pooled effect estimates or eliminate expert-selection bias. The framework should therefore be considered a testable study-design and longitudinal-monitoring tool that complements, rather than replaces, antiviral development and validated virus-specific clinical algorithms.

## Figures and Tables

**Table 1:** Comparative endothelial and hemostatic features of SARS-CoV-2, dengue-centered flavivirus evidence, and hantaviruses across ten prespecified indicators, with the evidence level stated for each entry. The table is a qualitative, disease-constrained comparison rather than a quantitative grade. Mixed phenotypes are represented by retaining more than one active program—for example, severe dengue with both plasma leak and increased thrombin generation—and by interpreting each entry in relation to disease phase and treatment exposure.

Indicator	SARS-CoV-2	Dengue (with Notes on Other Flaviviruses)	Hantaviruses
**Direct endothelial infection**	**Context-dependent**—Endothelial-associated viral RNA or protein has been reported, but widespread productive infection is not consistently demonstrated; indirect activation predominates [1,3,34,35].	**Context-dependent**—Endothelial infection is reported for selected viruses and experimental models, but is not uniform across the genus and is not required to explain DENV vascular leak [47,55,56].	**Established**—Endothelial tropism is well-supported and is predominantly non-lytic in pulmonary and renal microvascular beds [17,19,21].
**Endothelial activation after infection**	**Established**—Marked endothelial activation and endotheliopathy occur in severe disease [2,4,5].	**Established** for severe DENV; context-dependent across non-DENV flaviviruses and specialized vascular barriers [45,46,47,55,56,57,58].	**Established**—Endothelial activation and dysfunction are prominent in the renal and pulmonary microvascular beds affected in HFRS and HCPS [17,21,61].
**Inflammation intensity**	**Established**—High systemic and pulmonary thromboinflammation in severe disease, involving cytokines, complement, and NETosis [2,8,9,10,27].	**Established** for severe DENV, but variable by virus, immune history, and disease stage [15,16,47].	**Established**—Strong antiviral and inflammatory responses accompany severe HFRS and HCPS, with organ-weighted expression [17,18,61].
**Endothelial structural damage**	**Context-dependent**—Endothelial injury is documented, but direct endothelial cytolysis is not a uniform finding [1,2,3,4,34,35].	**Established** for functional glycocalyx and barrier disruption in DENV models; overt endothelial cytolysis is limited and context-dependent [12,13,14,48].	**Established**—Severe leakage occurs with limited cytolysis and relative preservation of endothelial structure [17,21,61].
**Mechanism of increased permeability**	**Context-dependent**—Inflammation, complement activation, glycocalyx loss, and junctional dysfunction contribute, especially in pulmonary microvasculature [2,27,32].	**Established** in DENV models—NS1-mediated glycocalyx disruption acts with inflammatory and junctional mechanisms [12,13,14,48]. **Context-dependent** for non-DENV flaviviruses.	**Context-dependent**—Integrin/VEGF signaling, immune mediators, junctional changes, and kallikrein-kinin activation vary by species and model [18,19,21,62].
**Coagulation activation**	**Established**—Severe disease is characterized by high D-dimer, increased thrombin generation, high fibrinogen and factor VIII/vWF, and impaired fibrinolysis; falling fibrinogen and prolonged PT/aPTT generally indicate advanced critical illness [8,31,38,39].	**Context-dependent**—Severe dengue may show prolonged PT/aPTT, reduced fibrinogen or coagulation factors, increased fibrin degradation, and measurable thrombin generation; severe YFV additionally involves hepatic factor depletion and consumption, whereas coagulation activation is generally less prominent in neurotropic flaviviruses [53,54,57]. **Hypothesis-generating**—Non-DENV NS1 effects on tissue factor-bearing microvesicles remain unreplicated [52]. A low platelet count may coexist with platelet activation and a prothrombotic state and should be interpreted, when available, together with immature platelet fraction and platelet-function or aggregate assays.	**Context-dependent**—HFRS/HCPS may show elevated D-dimer, prolonged clotting times, thrombocytopenia and phase-dependent changes in fibrinogen or thrombin generation, although capillary leak usually dominates [59,60,61]. A low platelet count does not by itself establish hypocoagulability and should be interpreted with disease phase, thrombin markers, and platelet-function or aggregate assays.
**Extent of thrombosis**	**Established**—Microvascular and macrovascular thrombosis are prominent complications [1,8,31].	**Context-dependent** and generally limited—Thrombosis is not the dominant phenotype, although microvascular and coagulation abnormalities may occur in severe disease [53,54,57].	**Context-dependent** and usually limited—Macrovascular thrombosis is not defining; bleeding and thrombotic risks vary with disease phase and organ involvement [59,60,61].
**Range of organ involvement**	**Established**—Lung and systemic vasculature predominate, with cardiac, renal, and neurological involvement in severe disease [1,2,3,4].	**Established** but virus-specific—Systemic vasculature in DENV, placenta and CNS in ZIKV, CNS in WNV/JEV/TBEV/USUV, and liver in YFV [45,46,47,48,49,52,53,54,55,56,57,58].	**Established**—Predominantly kidney in HFRS and lung in HCPS, with clinical overlap [59,60,61].
**Endothelial activation markers**	**Established**—Increased vWF, soluble thrombomodulin, E-selectin, and angiopoietin-2 [4,70]. **Hypothesis-generating**—PROS1/protein S and HLA-G/NK/KIR modifiers [33,36,37].	**Context-dependent**—vWF, angiopoietin-2, and glycocalyx or permeability markers vary with timing, virus, and cohort [48,50,54].	**Context-dependent**—vWF, angiopoietin-2 and permeability-related markers have been reported, but assays and timing are not standardized [17,61].
**Pathological characteristics**	**Established**—Diffuse alveolar damage, endotheliopathy, microvascular thrombosis, and angiogenic remodeling characterize severe disease [1,2,3].	**Established** for severe DENV—Plasma leakage, thrombocytopenia, and hemorrhagic manifestations occur with limited cytolysis; other flaviviruses show organ-specific placental, neurovascular, or hepatic pathology [45,46,47,48,49,52,53,54,55,56,57,58].	**Established**—Pulmonary edema or renal microvascular injury, interstitial edema, and hemorrhagic features occur with limited endothelial destruction [59,60,61].

Notes: Evidence labels apply to the specific claim in each cell, not to an entire virus group. Established = supported by multiple independent clinical, pathological, or mechanistic studies; context-dependent = reproduced in selected species, cohorts, disease stages, vascular beds, or models, but not broadly generalizable; hypothesis-generating = derived from pilot studies, limited cohorts, indirect associations, or specialized models. DENV NS1 and hantavirus VEGF/integrin or kallikrein–kinin pathways are disease-specific and model-dependent. The PROS1/protein S and HLA-G/NK/KIR-HLA observations [33,36,37,71] and the non-DENV NS1 microvesicle findings [52] are exploratory, group-derived, and currently unreplicated; they do not inform Figure 2 weighting or clinical recommendations. DENV NS1 findings should not be generalized to other flaviviruses.

**Table 2:** Candidate biomarkers for clinical and translational studies of viral vascular disease.

Pathway	Candidate Markers	Potential Interpretation	Caveats
**Endothelial activation/injury**	vWF, soluble thrombomodulin, E-selectin, angiopoietin-2	Endothelial activation, Weibel–Palade body release, vascular stress	Not specific to a single virus; influenced by age and comorbidities
**Glycocalyx/barrier integrity**	Syndecan-1, heparan sulfate fragments, albumin leakage, hematocrit dynamics	Barrier disruption and plasma leakage	Assay availability and timing are major limitations
**Coagulation/fibrinolysis**	D-dimer, fibrinogen, PT/aPTT, thrombin generation, FXa generation, thrombin–antithrombin complexes, prothrombin fragment 1 + 2, PAI-1, tissue factor-bearing vesicles.	Thrombin generation, fibrin turnover, fibrinolytic shutdown or hyperfibrinolysis, net hemostatic balance	D-dimer is sensitive but nonspecific; global and viscoelastic assays are poorly standardized across viral cohorts and are influenced by anticoagulant exposure, inflammation and sampling time
**Platelets**	Platelet count, immature platelet fraction, P-selectin, platelet–leukocyte aggregates	Consumption, activation, production response	Platelet count alone may misclassify risk
**Complement**	C3a, C5a, sC5b-9, factor Bb	Complement amplification and endothelial crosstalk	Pre-analytical handling is critical
**NETosis**	Cell-free DNA, MPO-DNA complexes, citrullinated histone H3	Neutrophil-driven thromboinflammation	Assays are not standardized for routine practice
**Protein S/anticoagulant pathway**	PROS1; free/total protein S; APC/protein S function	Exploratory marker of endogenous anticoagulant control and host vascular risk in selected subgroups	Affected by acute phase, anticoagulants, liver function and vitamin K status; not validated as a universal viral vascular biomarker

**Table 3:** Translational use of the four vascular programs for study design, phenotype-guided stratification, and candidate therapeutic or management implications.

Dominant Program	Candidate Clinical/Biomarker Signals	Interpretive Use	Caution	Therapeutic or Management Implication
**Endothelial activation/thromboinflammation**	High vWF or angiopoietin-2, platelet–leukocyte aggregates, NET markers, complement activation, elevated D-dimer	Identify patients or models in which immunothrombosis and endothelial activation are central outcomes	May reflect nonspecific critical illness; requires timing and comorbidity adjustment. Anticoagulant exposure and invasive organ support can dominate D-dimer and thrombin-generation interpretation and must be modeled as time-varying confounders.	Assess for thrombosis and apply virus-specific prophylaxis or treatment standards. Anticoagulation intensity should follow validated indications and bleeding risk, not biomarker elevation alone; complement-, NET-, or endothelial-directed approaches remain investigational [40,41].
**Endothelial barrier failure/leak**	Hemoconcentration, albumin leakage, syndecan-1 or heparan sulfate fragments, tissue-specific edema, shock physiology	Prioritize glycocalyx, junctional integrity and vascular permeability endpoints	Leak may be transient and organ-specific; single time-point sampling can miss the window	Use carefully titrated fluid and hemodynamic support guided by intravascular volume and organ function. Avoid indiscriminate diuresis during active leak; glycocalyx- or permeability-directed agents remain investigational [45,46,61].
**Coagulation activation/impaired fibrinolysis**	D-dimer, fibrinogen, PT/aPTT, thrombin generation, PAI-1, tissue factor-bearing vesicles	Distinguish thrombin generation and fibrin turnover from platelet count alone	Bleeding risk and anticoagulant exposure must be incorporated	Evaluate for thrombosis and correct reversible procoagulant or bleeding factors. Initiate or intensify anticoagulation only when supported by disease-specific evidence, confirmed thrombosis or protocol-defined criteria [40,41].
**Platelet quantitative or qualitative dysfunction**	Platelet count, immature platelet fraction, P-selectin, platelet-derived vesicles, platelet–endothelial, or platelet–leukocyte interactions	Separate thrombocytopenia from platelet activation or consumption-driven vascular injury	Low platelet count can coexist with prothrombotic activation	Integrate platelet count with bleeding, activation and procedural risk. Avoid automatic platelet transfusion or antiplatelet therapy based on count or activation markers alone; follow virus-specific thresholds and indications [45,46,61].

**Table 4:** Ranked research priorities for validation of the vascular-program framework.

Priority	Research Objective	Proposed Approach and Readouts
**1**	Validate program assignment prospectively	Apply a shared four-time-point panel to aligned COVID-19, dengue and hantavirus cohorts, measuring vWF/angiopoietin-2, syndecan-1, D-dimer/TAT, and platelet–leukocyte aggregates.
**2**	Define vascular-bed specificity	Compare pulmonary, renal and glycocalyx-focused microvascular endothelial models using permeability, VE-cadherin, and thrombin-generation readouts.
**3**	Test the glycocalyx as a shared final pathway	Use a perfused organ-on-chip model with matched patient plasma and quantify syndecan-1 shedding and tracer leakage.
**4**	Resolve platelet state despite thrombocytopenia	Combine immature platelet fraction with platelet activation, aggregation, and leukocyte-binding assays.
**5**	Identify viral and host determinants that redirect the vascular phenotype	Perturb NS1, complement, NET and VEGF/kallikrein pathways in the appropriate organ-specific models.
**6**	Independently replicate hypothesis-generating findings	Validate the authors’ PROS1/HLA-G/KIR and non-DENV NS1 findings in preregistered external cohorts or independent laboratories before they influence framework weighting or translation [33,36,37,52,71]

## Data Availability

No new data were created or analyzed in this study. Data sharing is not applicable to this article.

## Supplementary Materials

The following supporting information can be downloaded at: https://www.mdpi.com/article/10.3390/v18101160/s1, Supplementary Methods S1, full database-specific search strategies and eligibility criteria; Supplementary Table S1, auditable evidence matrix underlying the expert schematic in Figure 2.
