# Supplementary material for: Vascular Programs in Emerging and Re-Emerging RNA Virus Infections: Comparative Lessons from SARS-CoV-2, Flaviviruses, and Hantaviruses
Source: Viruses. 2026 Oct 19;18(10):1160. doi: 10.3390/v18101160 (PMC13709913; doi:10.3390/v18101160)
Supplement: Supplementary file 1 [file viruses-18-01160-s001.zip › viruses-4551525-supplementary.pdf]

Supplementary Materials

Vascular Programs in Emerging and Re-emerging RNA Virus Infections

Supplementary Methods S1. **Full database-specific search strategies and eligibility criteria**. PubMed: ((“SARS-CoV-2”[Title/Abstract] OR “COVID-19”[Title/Abstract] OR dengue[Title/Abstract] OR flavivirus*[Title/Abstract] OR hantavirus*[Title/Abstract]) AND (endotheli*[Title/Abstract] OR “vascular leak”[Title/Abstract] OR coagulopath*[Title/Abstract] OR immunothrombo*[Title/Abstract] OR thromboinflamm*[Title/Abstract] OR platelet*[Title/Abstract] OR complement[Title/Abstract] OR NETosis[Title/Abstract] OR glycocalyx[Title/Abstract])). Web of Science Core Collection: TS=((SARS-CoV-2 OR COVID-19 OR dengue OR flavivirus* OR hantavirus*) AND (endotheli* OR “vascular leak” OR coagulopath* OR immunothrombo* OR thromboinflamm* OR platelet* OR complement OR NETosis OR glycocalyx)). Google Scholar: each virus term (“SARS-CoV-2”, dengue/flavivirus, hantavirus) was paired separately with each vascular term (endothelial dysfunction, vascular leak, coagulopathy, immunothrombosis, thromboinflammation, platelets, complement, NETosis, glycocalyx), and the first 200 relevance-ranked results per query were screened. Searches were last updated on 31 July 2026; English-language peer-reviewed publications were eligible, and preprints were excluded from evidence-tier assignment.

Supplementary Table S1. Auditable evidence matrix underlying the expert schematic in Figure 2.

Program × virus/model	Expert weight	Key evidence (study type; endpoints; references)	One-line rationale
**Endothelial activation — SARS-CoV-2**	Dominant	Human pathology and cohorts; vWF, thrombomodulin, angiopoietin-2, vascular inflammation [1-5, 11, 70]	Convergent clinical and pathological endotheliopathy with strong thromboinflammatory coupling.
**Endothelial activation — Dengue**	Moderate/context-dependent	Clinical cohorts plus experimental studies; cytokines, vWF, glycocalyx and junction markers [45-48, 50, 51]	Prominent in severe dengue, but timing, serotype and immune history modify expression.
**Endothelial activation — Hantaviruses**	Moderate/context-dependent	Human pathology/biopsies and endothelial models; microvascular activation and immune-cell recruitment [17, 21, 23, 61, 63-65]	Supported in pulmonary and renal beds, but species and organ context matter.
**Barrier failure — SARS-CoV-2**	Moderate/context-dependent	Human cohorts and mechanistic studies; glycocalyx loss, junctional dysfunction, pulmonary edema [2, 27, 32]	Leak occurs, especially in severe pulmonary disease, but is not the defining program.
**Barrier failure — Dengue**	Dominant	In vitro, animal and human cohorts; NS1, syndecan-1, junction markers, hemoconcentration [12-14, 48, 50, 51]	The most consistent program in severe dengue, while NS1 alone is insufficient for clinical leak.
**Barrier failure — Hantaviruses**	Dominant	Human pathology plus endothelial models; edema, preserved architecture, VEGF/integrin and FXII-kallikrein signaling [17, 19, 21, 23, 61-64]	Abrupt organ-specific capillary leak is the defining clinical output.
**Coagulation activation — SARS-CoV-2**	Dominant	Clinical cohorts, pathology and RCTs; D-dimer, thrombin generation, micro/macrothrombosis [8, 31, 38-41]	Replicated coagulation activation and thrombosis are central to severe disease.
**Coagulation activation — Dengue**	Weak/context-dependent	Clinical cohorts; PT/aPTT, fibrinogen, fibrin turnover; YFV hepatic coagulopathy [53, 54, 57]	Abnormalities occur in severe disease but leak, platelets and bleeding usually dominate.
**Coagulation activation — Hantaviruses**	Weak/context-dependent	Clinical series; D-dimer, clotting times and phase-dependent thrombin markers [59-61]	Coagulation changes occur, but evidence is less consistent than for capillary leak.
**Platelet dysfunction — SARS-CoV-2**	Moderate/context-dependent	Clinical/mechanistic cohorts; platelet-monocyte aggregates, activation and vesicles [5, 8]	Activation is reproducible, but severe quantitative thrombocytopenia is less typical.
**Platelet dysfunction — Dengue**	Dominant	Clinical cohorts and reviews; thrombocytopenia, activation, consumption and impaired function [29, 53, 54]	Quantitative and qualitative platelet abnormalities are defining and interact with leak.
**Platelet dysfunction — Hantaviruses**	Moderate/context-dependent	Clinical cohorts and endothelial models; early thrombocytopenia, adhesion/sequestration [29, 61, 67]	Early platelet loss is consistent, while net hemostatic effect varies by phase and organ.
